# Commentary: Estimating the effects of non-pharmaceutical interventions on COVID-19 in Europe

**DOI:** 10.3389/fmed.2020.580361

**Published:** 2020-11-05

**Authors:** Christof Kuhbandner, Stefan Homburg

**Affiliations:** ^1^Department of Human Sciences, University of Regensburg, Regensburg, Germany; ^2^Department of Public Finance, Leibniz University Hannover, Hannover, Germany

**Keywords:** SARS-CoV-2, COVID-19, non-pharmaceutical interventions, mitigation strategies, epidemiology

A key concept in epidemiology is the effective reproduction number, *R*(*t*), where *t* denotes time. This function represents the expected number of infections generated by one infected individual. Ceteris paribus, the effective reproduction number starts at *R*(0), referred to as the basic reproduction number, and decreases monotonically. The monotonic decrease is due to the fact that the number of individuals susceptible to the infection but not yet infected declines as the virus spreads. Of course, the function *R*(*t*) can be influenced by non-pharmaceutical interventions (NPIs) as well as by voluntary behavioral changes. However, in case of a finite population, the effective reproduction number falls automatically and necessarily over time since the number of infections would otherwise diverge. For recent discussions of the *R*(*t*), see (1, 2).

The model of Flaxman et al. (3) contradicts this elementary insight. They estimate *R*(*t*) from daily deaths associated with SARS-CoV-2 using as an *a priori* restriction that *R*(*t*) may *only* change at those dates where interventions become effective. Such an approach does not prove that NPIs were effective but rather begs the result, i.e., involves circular logic. The true effective reproduction number declines continuously, and when its estimates are allowed to change only at intervention points, it is clear that profound discontinuities, which attribute strong effects to the interventions, will emerge. Flaxman et al. (p. 2) conclude that while most NPIs had unidentifiable effects, lockdowns reduced the reproduction numbers instantaneously by 82%. Taking the United Kingdom as an example, Figure 1A illustrates the ineffectiveness of social distancing, etc. in the analysis of Flaxman et al. as well as the enormous effect of the lockdown from 23 March.

**Figure 1:**
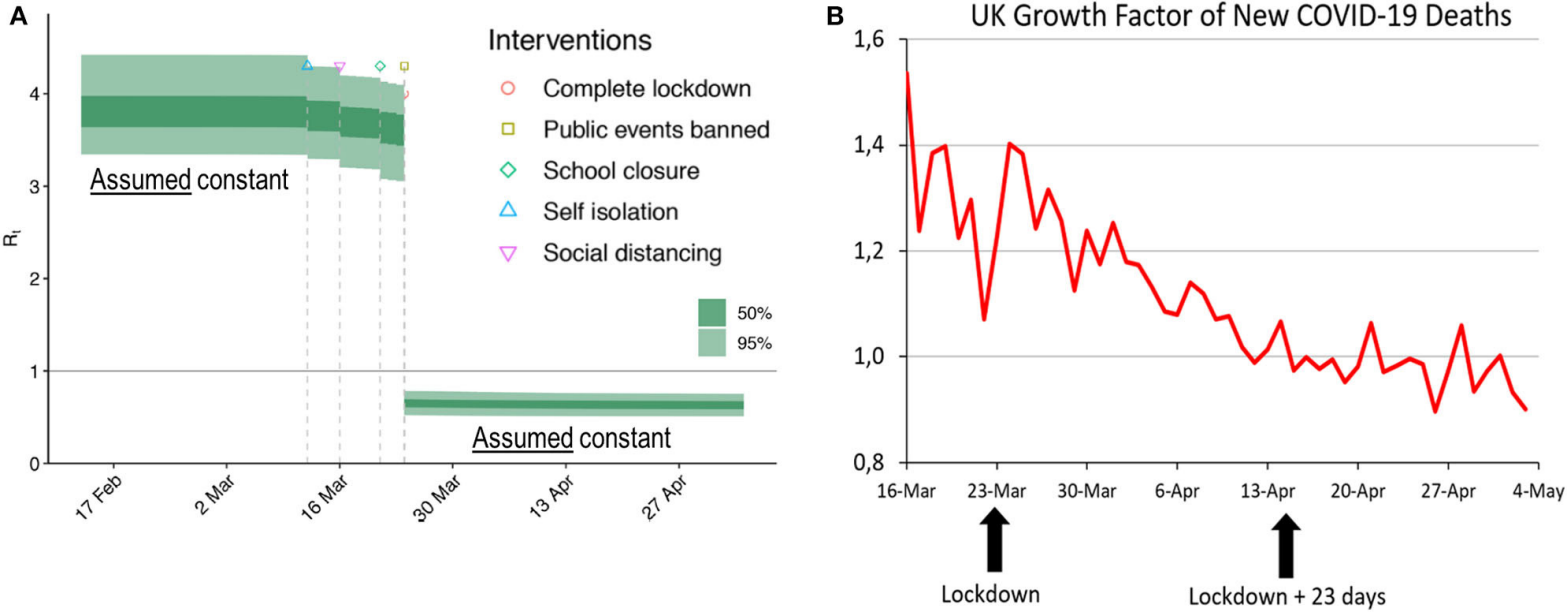
**(A)** Estimate of the effective reproduction number by Flaxman et al. [(3), Figure 1]. The authors assumed *R*(*t*) to be constant before 14 March and after 23 March. Changes were allowed only on the four dates where NPIs became effective. **(B)** Growth factor of daily deaths. Source: https://assets.publishing.service.gov.uk/government/uploads/system/uploads/attachment_data/file/891710/2020-06-11_COVID-19_UK_deaths_time_series.csv. Moving averages, 7 days. Retrieved 14 June 2020. Given daily deaths *d*_*t*_, growth factors were computed as *d*_*t*_/*d*_*t*−__1_. Note that if daily deaths show exponential growth, any moving average will also show exponential growth.

Flaxman et al. (p. 2) infer their estimate of the basic reproduction number from the initial growth of reported daily deaths. Figure 1B shows the actual growth of reported daily deaths. In both cases, the growth factors considered are defined as *d*_*t*_/*d*_*t*−__1_, where *d*_*t*_ represents the number of fatalities reported for day *t*. Following the presumption of Flaxman et al. that deaths are more reliable than cases, we see the growth factors of daily deaths as a good empirical proxy that mirrors the development of the effective reproduction rate. Of course, deaths follow infections after a long delay—a fact that is taken into account below.

Disregarding noise in the data, the growth in daily deaths associated with the coronavirus declined steadily during March and April. Moreover, reported daily deaths follow infections with a median delay of 23 days, consisting of a 5-day incubation period (4) and a median delay of about 18 days from symptom onset to death (5). Note that this delay also underlies the estimations by Flaxman et al. (p. 22 of their supplementary information). In a recent paper, Wood (6) estimates a longer delay of 26.8 days for UK. We do not use this estimate because it was not available for Flaxman et al. but note that taking account of the longer delay would even strengthen our point.

Considering a total delay of 23 days between infection and death, possible effects of the 23 March lockdown should only become visible in the data around April 15. However, the series does not show the slightest break in mid-April. Hitherto, the growth factor had already declined from 1.54 to 0.97, and thereafter it continued its slowdown. Quite contrary to the findings of Flaxman et al. Figure 1B strongly suggests that the UK lockdown was both superfluous (it did not prevent an otherwise explosive behavior of the spread of the coronavirus) and ineffective (it did not slow down the death growth rate visibly).

The argument of a delay of 23 days between infection and death can also be used in the opposite direction. With the growth rate of daily corona deaths falling since mid-March, the underlying growth rate of daily infections must have started receding in the second half of February, long before the problem was recognized and any measures were taken. The continuous decrease in the growth factor shown in Figure 1B, even at dates before any NPI could have become effective, corroborates the theoretical insight that *R*(*t*) falls automatically over time. We have checked that the growth factors in the remaining 10 countries considered by Flaxman et al. show a similar pattern. Our analysis does not answer the question whether the decrease of *R*(*t*) was due to a decreasing number of susceptible persons or to voluntary behavioral changes, but it rules out the possibility that the decrease was caused by the general lockdown.

Our final remark regards Sweden, the only country in the dataset that refrained from strong measures, but has lower corona deaths per capita than Belgium, Italy, Spain, or the United Kingdom. In the absence of a lockdown, but with an effective reproduction number that declined in the usual fashion, Flaxman et al. (Extended Data Figure 1) attribute the sudden decline in Sweden's *R*(*t*) on March 27 almost entirely to banning of public events, i.e., to a NPI that they found ineffective in all other countries. This inconsistency underlines our contention that the results of Flaxman et al. are artifacts of an inappropriate model.

## Author Contributions

CK and SH co-wrote the paper.

## Conflict of Interest

The authors declare that the research was conducted in the absence of any commercial or financial relationships that could be construed as a potential conflict of interest.

## References

[1] Delamater PL Street EJ Leslie TF Yang Y Jacobsen KH Complexity of the basic reproduction number (R0). Emerg Infect Dis. (2019) 25:1–4. 10.3201/eid2501.171901 PMC6302597 30560777

[2] Li MY Important concepts in mathematical modeling of infectious diseases. In: Golden K, Lewis M, Nishiura Y, Sambridge M, Tribbia J, Zubelli JP, editors. An Introduction to Mathematical Modeling of Infectious Diseases. Mathematics of Planet Earth, Vol. 2 Cham: Springer (2018) p. 126–135. 10.1007/978-3-319-72122-4_1

[3] Flaxman S Mishra S Gandy A Unwin HJT Mellan TA Coupland H. Estimating the effects of non-pharmaceutical interventions on COVID-19 in Europe. Nature. (2020) 584:257–61. 10.1038/s41586-020-2405-7 32512579

[4] Lauer SA Grantz KH Bi Q Jones FK Zheng Q Meredith HR. The incubation period of coronavirus disease 2019 (COVID-19) from publicly reported confirmed cases: estimation and application. Ann Intern Med. (2020). 10.7326/M20-0504 32150748 PMC7081172

[5] Verity R Okell LC Dorigatti I Winskill P Whittaker C Imai N. Estimates of the severity of COVID-19 disease. Lancet Infect Dis. (2020) 20: 669–77. 10.1016/S1473-3099(20)30243-7 32240634 PMC7158570

[6] Wood SN. Did COVID-19 Infections Decline Before UK Lockdown? Available online at: https://arxiv.org/abs/2005.02090

[7] Kuhbandner C Homburg S Comment on Flaxman (2020): the illusory effects of non-pharmaceutical interventions on COVID-19 in Europe. Advance [Preprint]. Available online at: https://advance.sagepub.com/articles/preprint/Comment_on_Flaxman_et_al_2020_The_illusory_effects_of_non-pharmaceutical_interventions_on_COVID-19_in_Europe/12479987 10.3389/fmed.2020.580361 PMC7674856 33251231

